# Avelumab in newly diagnosed glioblastoma

**DOI:** 10.1093/noajnl/vdab118

**Published:** 2021-08-25

**Authors:** Francois H Jacques, Garth Nicholas, Ian A J Lorimer, Victorine Sikati Foko, Jasmine Prevost, Nathalie Dumais, Katy Milne, Brad H Nelson, John Woulfe, Gerard Jansen, B Erik Apedaile

**Affiliations:** 1 Clinique Neuro-Outaouais, Gatineau, Quebec, Canada; 2 Cancer Therapeutics Program, Ottawa Hospital Research Institute, Ottawa, Ontario, Canada; 3 Department of Biochemistry, Microbiology and Immunology, University of Ottawa, Ottawa, Ontario, Canada; 4 Department of Medicine, University of Ottawa, Ottawa, Ontario, Canada; 5 Deeley Research Centre, BC Cancer, Victoria, British Columbia, Canada; 6 Department of Biochemistry and Microbiology, University of Victoria, Victoria, British Columbia, Canada; 7 Department of Medical Genetics, University of British Columbia, Vancouver, British Columbia,Canada; 8 Department of Pathology and Laboratory Medicine, University of Ottawa, Ottawa, Ontario, Canada

**Keywords:** avelumab, glioblastoma, immune checkpoint inhibitor, PD-L1, phase II

## Abstract

**Background:**

Glioblastoma (GBM) is known to use both local and systemic immunosuppressive strategies. One such strategy is the expression of the immune checkpoint protein programmed cell death ligand-1 (PD-L1) by both tumor cells and tumor-associated immune cells. Recent phase III trials using IgG4 antibodies targeting PD-1, the ligand for PD-L1, failed to show any benefit. Avelumab is an IgG1 monoclonal antibody targeting PD-L1. In contrast to the previously tested immune checkpoint inhibitors, it can directly bind tumor cells and immune cells expressing PD-L1 and can induce antibody-dependent cellular cytotoxicity.

**Methods:**

We conducted a single center, open label, phase II study where avelumab 10 mg/kg IV Q2W was added concurrently to the first monthly temozolomide cycle in patients with newly diagnosed GBM. Immunohistochemical analyses were performed on surgery samples. The primary objective was safety. Secondary objectives were efficacy outcomes according to the immunotherapy Response Assessment in Neuro Oncology criteria, progression free survival (PFS), and overall survival (OS). Exploratory objectives aimed at determining prognostic biomarkers.

**Results:**

Thirty patients were started on therapy and two were lost to follow-up. Median follow-up time (reverse Kaplan-Meier) was 41.7 months (IQR: 28.3–43.4). Three (10.0%) patients had a related or possibly related treatment emergent adverse event that lead to transient or permanent discontinuation of avelumab. Eight (26.7%) patients had one or more immune-related adverse events, and 8 (26.7%) patients had an infusion-related reaction. The overall response rate was 23.3%, median PFS was 9.7 months, and the median OS was 15.3 months. No pretreatment biomarkers showed any predictive value.

**Conclusions:**

The addition of avelumab to standard therapy in patients with GBM was not associated with any new safety signal. There was no apparent improvement in OS.

**Trial Registration:**

NCT03047473 Registered February 9, 2017.

**Key Points**

- The combination of avelumab with temozolomide in newly diagnosed glioblastoma patients is safe.
- With the assays used in the study, none of the biological markers assessed in pretreatment GBM tumor tissue samples had any associated predictive value.
- New therapeutic combinations will be required to enhance the immunotherapeutic effect in GBM patients.

**Importance of the Study**

GBM patients treated with a combination of radiotherapy and temozolomide have a median survival of 15 months.^1^ Previous trials using immunotherapies in GBM patients have failed to show a significant survival benefit. This is the first clinical study combining an anti-PD-L1 immunotherapy with combination radiotherapy/temozolomide in newly diagnosed GBM patients. The study demonstrated the safety profile of such combination and approach with an objective response rate of 23% and progression free survival of 9.7 months. The overall survival however remained at 15.3 months. The study confirmed observations from other studies regarding the persistent use of steroids early on as a negative prognostic factor and the lack of predictive value of pretreatment tumor tissue immunohistochemical analyses including PD-L1. Analysis of on-treatment tumor tissue in a small group failed to show any significant treatment effect. The study is ongoing to determine the extent of benefit in a subgroup of potential responders.

Glioblastoma (GBM) is the most common malignant primary brain tumor in adults.^2^ Without treatment, the 1- and 5-year survival is 29% and 3%, respectively.^3^ Methyl-guanine methyl transferase (MGMT) promoter methylation in patients with GBM (35% incidence) is associated with better overall survival (OS).^4,5^ Mutations in the isocitrate dehydrogenase gene (IDH) 1 or 2 (5%–10% incidence) are also associated with a better prognosis independent of MGMT methylation.^6^ With standard treatment of maximum safe resection followed by concurrent radiotherapy (60 Gy) and temozolomide (TMZ) followed by six monthly cycles of TMZ, the median survival is 15 months and the 2-year survival 27%.^1^ Trials looking at escalating the radiotherapy dose beyond the 60 Gy, increasing the dose of TMZ as adjuvant or maintenance therapy^7^ or the addition of cilengitide^8^ or bevacizumab,^9^ have failed to show any improvement in OS.

GBMs are known to enact both systemic and local immunosuppressive strategies to evade the host immune system and enhance tumor progression. They secrete systemic factors that act to decrease T- and B-cell responsiveness, cause lymphopenia of CD4+ T cells and NK cells, increase the fraction of Treg cells, reduce immunoglobulin production, and increase numbers and activity of immunosuppressive myeloid cells, including myeloid-derived suppressor cells and immunosuppressive microglia/macrophages.^10,11^ GBM can immunosuppress within its microenvironment creating a perimeter of immune defense by producing cytokines such as transforming growth factor β,^12^ interleukin 2, 6, and 10,^13,14^ and prostaglandin E.^14^ The microglia, which can comprise up to 40% of the tumor mass, acquire an immunosuppressive M2 phenotype, expressing proteins such as matrix metalloproteinase 9, vascular endothelial growth factor, and IL-10. Both glioma cells and glioma-associated microglia also express programmed cell death ligand-1 (PD-L1) and other immune checkpoint regulators. These immunosuppressive surface molecules downregulate the antitumor functions of immune cells such as cytotoxic CD8+ T and NK cells by inducing their anergy or apoptosis.^10,11^ Glioma tumor grade has been correlated with PD-L1 expression and degree of Treg infiltration.^15–17^ These data suggest an important role for PD-L1 in GBM immunosuppression, and there is considerable interest in targeting PD-L1, or its ligand PD-1, in this disease.

While antibodies targeting the PD1/PD-L1 immune checkpoint have shown remarkable success in some cancers, to date they have not shown a survival benefit in large phase III studies in GBM.^18^ However, two recent small trials in which anti-PD1 antibodies were given shortly before second surgeries showed promising immunomodulatory effects^19,20^ and, in one trial, a small survival benefit.^20^ For PD1/PD-L1 immune checkpoint targeting, the anti-PD1 antibodies, nivolumab and pembrolizumab, have been most widely studied. Both antibodies are IgG4, which are predicted to be unable to induce antibody-dependent cellular cytotoxicity (ADCC) as they do not bind Fcγ receptors. In the case of nivolumab, it has been directly shown that it is unable to mediate either antibody or complement-dependent cytotoxicit.^21^ The human monoclonal antibody avelumab also targets the PD1/PD-L1 immune checkpoint but binds PD-L1 rather than PD1. Like nivolumab and pembrolizumab, this promotes T-cell activation by antagonizing the PD1/PD-L1 axis. As targeting PD-L1 removes the concern of killing beneficial activated T cells, avelumab has been made as an IgG1, which is able to bind Fcγ receptors to mediate ADCC. Avelumab may therefore have additional mechanisms of action, including ADCC-mediated killing of either cancer cells overexpressing PD-L1 or other PD-L1 expressing immunosuppressive cells. While ADCC is traditionally thought to be mediated by NK cells, phagocytic cells also express Fcγ receptors and are capable of antibody-dependent cell killing. Both blood-derived macrophages and microglia, the tissue-resident macrophages of the brain,^22^ can function in this capacity. It is therefore possible that avelumab, an IgG1 anti-PD-L1, may have more beneficial impact on GBM than IgG4 anti-PD1 monoclonal antibody therapies. We conducted a phase II study to determine the safety profile and potential survival benefit of avelumab when added to standard therapy in newly diagnosed GBM patients.

## Materials and Methods

### Study Design

We conducted a single center, phase II, open label, add-on study in patients receiving standard therapy for newly diagnosed GBM. Ethics approval was received from the Canadian SHIELD Ethics Review Board, Registration No. 94025 February 23, 2017, renewed annually to February 22, 2022. In total, 30 patients were entered into the study within 3 weeks of finishing their last day of combined radiotherapy/TMZ. Informed consent was obtained from each patient in the presence of a family member prior to any study related procedure. Avelumab 10 mg/kg IV was initiated concurrently with the initiation of the first postradiotherapy cycle of TMZ. Avelumab was administered every 2 weeks thereafter throughout the 6 months of TMZ and subsequently as monotherapy until confirmation of tumor progression according to the immunotherapy Response Assessment in Neuro Oncology (iRANO) criteria or the occurrence of an end of therapy event. The latter was defined as either clinical evidence of neurological deterioration resulting in an Eastern Cooperative Oncology Group performance status scale (ECOG) score of at least 3 or more (unexplained by other comorbidities, unchanged by an increase in corticosteroid dose, and sustained for at least 2 weeks) or treatment emergent adverse event (TEAE) of grade 3 or more or withdrawal of patient consent. Upon confirmation of disease progression, patients were offered continuation of the avelumab therapy as monotherapy or in combination with other therapies or to withdraw from avelumab therapy and enter an Extended Safety Follow-up phase. The use of bevacizumab, a second debulking, or reirradiation was not allowed as a combination with avelumab in the study. MRI or CT scans performed every 3–4 months according to local standard of care and every 12 months at our center were evaluated by a single study radiologist.

The primary endpoint was safety and tolerability based on avelumab TEAEs leading to permanent or transient discontinuation of avelumab. Immune-related adverse events (irAEs) were considered TEAEs of special interest. Adverse events were graded according to the National Cancer Institute Common Terminology Criteria for Adverse Events, version 5.0. Secondary endpoints included the clinical and radiological tumor responses according to the iRANO criteria as well as progression free survival (PFS) and OS. Survival times were determined from the date of diagnosis and compared to Stupp et al.^1^ Exploratory endpoints included the correlation between OS and PFS and biomarkers in tumor tissue obtained at diagnosis, the change in those markers in tumor tissue obtained at a second debulking (in patients who continued in the survival follow-up) as well as correlations with the concomitant use of corticosteroids, P300 evoked potentials obtained at baseline, and the incidence of irAEs.

### Statistical Analyses

All statistical analyses were performed using R on the basis of intention to treat. Paired groups were compared using Fisher’s Exact Test for categorical variables and a *t* test for continuous variables. Multiple comparisons were performed using analysis of variance and Tukey HSD. Survival analysis including Kaplan-Meier plots and Cox proportional hazard calculations were performed using the survivalAnalysis and ggplot2 packages in R.^23–25^

### Immunohistochemical Analyses

The following antibodies were used: CD8 (cat# 108M-94, clone# C8144B) and PD-1 (cat# 315M-95, clone# NAT105) from Cell Marque; CD3 (cat# M3074, clone# SP7), PDL1(cat# M4422, clone# SP142), and CD68 (cat# M5510, clone# SP251) from Spring Bioscience/Abcam; CD20 (cat# CM004, clone# L26) from Biocare Medical/Inter Medico; and PTEN (cat# 9559, clone# 138G6) from Cell Signaling Technologies. Multicolor immunohistochemistry for CD3/CD8/CD20 and PD1/PDL1/CD68 panels was performed using a Biocare Intellipath FLX autostainer. CD8 and CD3 antibodies were used together, followed by Mach2 Double Stain 2 polymer; for the other panel, PD1 and PDL1 antibodies were used together followed by Mach2 Double Stain 1. Slides were then developed with Ferangi Blue and 3,3′-diaminobenzidine chromogens. Slides were then stripped of the first-round antibodies and incubated with the third antibody in the panel followed by either Mach 2 Mouse-AP polymer (for CD20) or Mach 2 Rabbit-AP polymer (for CD68). Slides were then developed with Warp Red chromogen and stained with hematoxylin. Slides were imaged using a Vectra 3 multispectral imaging system (Akoya Biosciences). Images were spectrally separated and analyzed using in inForm imaging software (Akoya Biosciences). For the analysis, three algorithms were generated for each panel, training the inForm software on viable tissue/necrosis/blank space from 10 images from the dataset. InForm software was then trained to find nuclei and surrounding staining. Cell phenotypes were then defined and inform was retrained until its output matched visual analysis. Algorithms were then run against all the images for the panel. Data were processed for display in Excel using Spotfire (Perkin Elmer). InForm output data were validated by comparison with visual assessment on a subset of randomly selected images. Final data were expressed as positive cells per mm^2^ of viable tissue. Standard immunohistochemistry was performed for PTEN expression, and scoring was performed by two neuropathologists (J.W. and G.J.) using a 0 (no detectable PTEN), 1 (weak PTEN expression), or 2 (PTEN expression equivalent to normal tissue) scoring system. All immunohistochemical analyses were performed blinded to clinical outcomes. Further details of the immunohistochemistry and analysis procedures are available on request.

## Results

The first patient visit was in March 2017, the last patient enrolled in September 2019, and the analysis was initiated in November 2020. The median follow-up time (reverse Kaplan-Meier^26^) was 41.7 months (IQR: 28.3–43.4). The median time from diagnosis to start of combination radiotherapy/TMZ was 44 days (18–82 days) and to avelumab was 93.5 days (72–147 days). Patient demographics and baseline characteristics are detailed in Table 1.

**Table 1.** Patient Demographics and Characteristics at Baseline

Characteristic		n = 30
Age (y): median (range)		55 (31–74)
Age: no. (%)	<50 y	6 (20.0)
	≥50 y	24 (80.0)
Sex: no. (%)	Female	13 (43.0)
	Male	17 (57.0)
Surgery: no. (%)	Biopsy	6 (20.0)
	Debulking	24 (80.0)
IDH1 mutation: no. (%)	Present	2 (6.7)
	Absent	27 (90.0)
	Unknown	1 (3.3)
MGMT promoter methylation status: no. (%)	Methylated	10 (33.3)
	Unmethylated	16 (53.3)
	Unknown	4 (13.3)
Diagnosis to avelumab (days): median (range)	Median	93.5 (72–147)
Karnofsky score: median (range)	Median	90 (70–100)
ECOG: no. (%)	0	19 (63.3)
	1	9 (30.0)
	2	2 (6.7)
Tumor location at baseline: no. (%)	Basal ganglia/corpus callosum	5 (16.7)
	Lobar	24 (80.0)
	Both	1 (3.3)
Tumor characteristics at baseline	Nonmeasurable tumor	13
	Single lesion	24
	Multiple lesions	6
	SPD^a^ (cm^2^): median (range) (17 patients with measurable tumor)	4.95 (1.4–25.8)
	T2 FLAIR (cm^2^): median (range) (all 30 patients)	19.3 (3.2–57.6)

ECOG, Eastern Cooperative Oncology Group performance status scale. ^a^Sum of the product of biperpendicular diameters.

### Study Evolution

Thirty-eight patients were screened and 30 were enrolled. Reasons for screening failure were withdrawal of consent (four patients), laboratory abnormalities (three patients), and low Kanorfsky score (one patient). Out of the 30 patients enrolled, 2 were lost to follow-up, 26 have shown tumor progression according to iRANO criteria, and 2 remain in complete response and on avelumab therapy. Of those who progressed, seven underwent a second debulking, one received further radiotherapy, and eight received salvage medical therapy, which included lomustine, axitinib, avelumab, or bevacizumab, alone or in combination. In total, seven remain alive at the time of analysis, of which four are continuing avelumab therapy.

### Safety and Tolerability

The majority of TEAE that led to transient or permanent avelumab discontinuation were related to tumor progression. Three of them were related or possibly related to avelumab treatment (one case of meningitis and two cases of elevated liver enzymes). Eight (26.7%) patients developed one or more irAEs of which elevated liver enzymes were the most common (61.3%) and 22.6% were grade 3 or higher. Eight (26.6%) patients had an infusion-related reaction of which only one (syncope) was grade 3. No patient had to stop avelumab therapy because of an infusion-related reaction (Table 2).

**Table 2.** Adverse Events

CTCAE Term	All Grades: No. (% of Patients) (n = 30)	Grades 3+: No. (% of Patiens) (n = 30)
Adverse effects that led to temporary or permanent avelumab discontinuation		
Disease progression	19 (63.3)	17 (56.7)
Radiation necrosis	2 (6.7)	2 (6.7)
Thromboembolic event	2 (6.7)	2 (6.7)
Seizure	2 (6.7)	2 (6.7)
COPD exacerbation	1 (3.3)	1 (3.3)
Rash NOS	1 (3.3)	0
Erythema multiform	1 (3.3)	1 (3.3)
Platelet count decreased	1 (3.3)	1 (3.3)
Fever	1 (3.3)	0
Hypotension	1 (3.3)	0
Urinary tract infection	2 (6.7)	2 (6.7)
Fatigue	1 (3.3)	0
Meningitis	1 (3.3)	1 (3.3)
AST elevated	3 (6.7)	0
GGT elevated	3 (6.7)	3 (6.7)
LDH elevated	2 (6.7)	0
ALT elevated	1 (3.3)	1 (3.3)
Alkaline phosphatase increased	1 (3.3)	0
Localized edema	1 (3.3)	1 (3.3)
Pneumatosis intestinalis	1 (3.3)	1 (3.3)
Infusion-related reactions		
Fever	5 (16.7)	0
Diarrhea	2 (6.7)	0
Nausea	2 (6.7)	0
Chills	1 (3.3)	0
Headache	1 (3.3)	0
Hypertension	1 (3.3)	0
Vomiting	1 (3.3)	0
Syncope	1 (3.3)	1 (3.3)
Immune-related adverse events		
Colitis	2 (6.7)	0
ALT increased	3 (6.7)	1 (3.3)
AST increased	5 (10.0)	0
LDH increased	5 (13.3)	0
GGT increased	4 (6.7)	4 (6.7)
Alkaline phosphatase increased	1 (3.3)	0
Hypophysitis	2 (6.7)	0
Hypothyroidism	2 (6.7)	1 (3.3)
Lipase Increased	1 (3.3)	0
Serum amylase increased	3 (6.7)	1 (3.3)
Mastocytosis	1 (3.3)	0
Rash	1 (3.3)	0

### Secondary Outcomes

**Figure 1.**
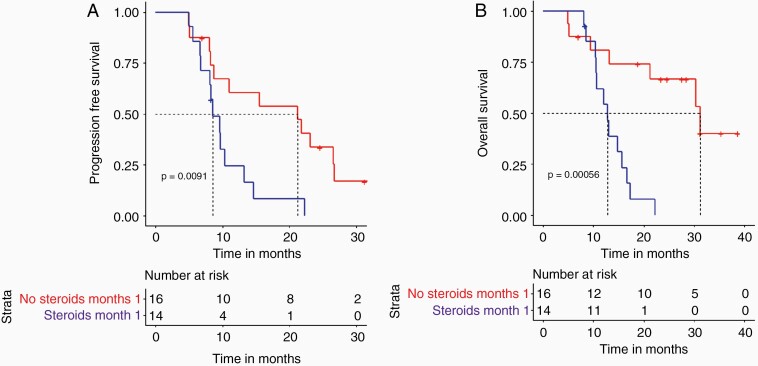
Kaplan Meier plots for patients grouped according to steroid use. A. progression-free survival in patients who did not require the use of steroids at month 1 (red) and those who did (blue). B. Overall survival in patients who did not require the use of steroids at month 1 (red) and those who did (blue).

Using iRANO criteria, the overall response rate was 23.3% with a median duration of response of 12 months (6.6–13.4 months). At 12 months from baseline, 11 (36%) patients were stable or showed evidence of treatment response (Table 3). The median PFS was 9.7 months (8.2–15.5) and median OS was 15.3 months (10.7–21.5). The percentage of patients with PFS at 6, 12, 24, and 30 months was 83%, 40%, 17%, and 7%, respectively, and the OS was 90%, 67%, 30%, and 23%, respectively (Table 4). Subgroup analysis of responders versus nonresponders showed a median PFS of 26.5 months (21.8–32.7) versus 8.3 (3.2–22.3) (*P* ≤ .001) and a median OS of 30.4 months (22.8–37.8) versus 12.8 (3.2–38.6) (*P* ≤ .001). Comparative analysis of the baseline clinical and radiological characteristics of the two groups was unable to discern any significant difference except for a higher incidence of IDH1+ in the responder group (two patients) (*P* = .05). When correlating individual baseline clinical and radiological characteristics of the whole group to PFS and OS only MGMT+ showed a significant correlation with PFS (*P* = .033) but not with OS. It is of note that neither the extent of surgical intervention nor the presence of irAE was associated with any survival advantage. Baseline use of steroids, the total cumulative dose, or the total duration of steroids did not show any correlation with outcome. The ongoing use of steroids at months 1 and 2, however, identified a group with a significantly different outcome. Patients who did not require the use of steroids at month 1 had a median PFS of 18.4 months versus 8.5 months (*P* = .02) (Figure 1A) and median OS of 22.6 months versus 12.4 months (*P* = .01) (Figure 1B). Comparative analysis between the steroid nonusers at months 1 and 2 and the users revealed a higher incidence of MGMT+ in the nonuser group (50% versus 14%) (*P* = .03). No other significant difference was found. Median postprogression survival was 3.7 months (0–23.1 months). Excluding patients who died within 1 month of having progressed, the median survival is 6.6 months (1.2–23.1 months).

**Table 3.** Assessment iRANO Response

iRANO	12 Months: n (%)	18 Months: n (%)	24 Months: n (%)
Complete response	4 (13.3)	5 (16.7)	2 (6.7)
Partial response	2 (6.7)	0 (0)	0 (0)
Stable disease	3 (10.0)	0 (0)	0 (0)
Disease progression	19 (63.3)	23 (76.7)	26 (86.7)

**Table 4.** Progression Free and Overall Survival

Interval	PFS		OS	
	(n)	(%)	(n)	(%)
6 months	25	83.3	27	90.0
12 months	12	40.0	20	66.7
18 months	9	30.0	12	40.0
24 months	5	16.7	9	30.0
30 months	2	6.7	7	23.3
36 months	0	0	3	10.0
Median survival (months)	9.7		15.3	

### Biomarker Analyses

**Figure 2.**
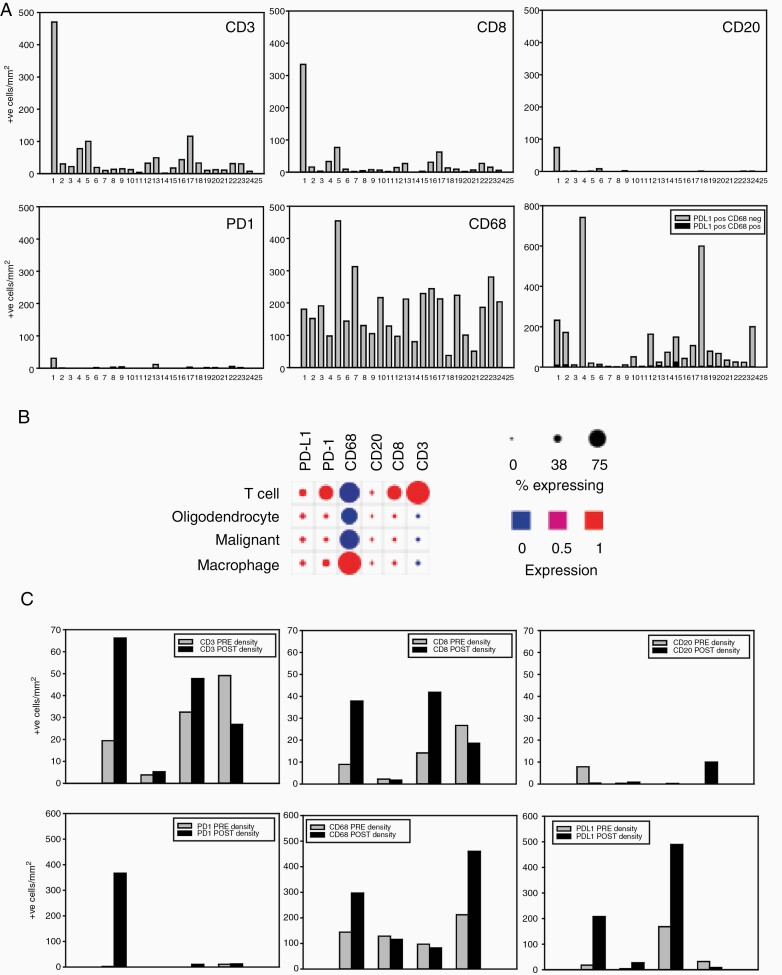
Biomarker analyses: A. Multicolour immunohistochemistry analyses of first surgery samples from trial patients. Top row of bar graphs show the results for the CD3/CD8/CD20 analysis. The bottom row of bar graphs show results for PD1/PDL1/CD68 analysis. Y axes show the number of positive cells per mm2 and x axes show results for individual patients. B. Single cell RNA-seq expression data from twenty-eight glioblastoma patients sequenced by Neftel et al.^3^ were analyzed for mRNA expression of immunohistochemistry markers used in this study, using Broad Institute Single Cell portal software https://singlecell.broadinstitute.org/single_cell. C. Comparison of immune status in first and second surgery samples from trial patients. Y axes show the number of positive cells per mm2 and x axes show paired bars for individual patient samples from first (grey bar) and second surgeries (black bar).

Tumor tissue samples from diagnostic surgeries or biopsies were obtained for 24 patients; for four of these patients’ samples from a second debulking were also obtained. Single sections were analyzed for either CD3/CD8/CD20 expression or PD-1/PD-L1/CD68 expression using multicolor immunohistochemistry.^27^ The top panels in Figure 2A show the results of analyses for CD3/CD8/CD20 in first surgery samples. CD3 positive cell numbers were, with the exception of one patient, generally low, as expected for GBM.^28^ Overall 50% of CD3 positive cells were positive for CD8 (range 13%–88%) while CD20+ B cells were either low or absent. This is consistent with mRNA expression data from single cell RNA-seq^29^ (Figure 2B). The bottom panels in Figure 2A show the results of analyses for PD-1/CD68/PD-L1. PD-1 positive cells were generally very low. As expected, based on the well-known extensive infiltration of GBMs with microglia/macrophage, CD68 positive cells were common. There was substantial variation between patients (range 38–454 cells per mm^2^); this is also expected given the known differences in microglia/macrophage engagement between GBM molecular subtypes.^30^ Numbers of PD-L1 positive cells were also very variable between patients (range 2–742 cells per mm^2^). While some microglia/macrophages were PD-L1-positive, most of the PD-L1 positive cells were CD68 negative and likely represent GBM cells. Previous studies have given conflicting estimates of PD-L1 expression in GBM.^31,32^ Analysis of single cell RNA-seq data generated by Neftel et al.^29^ shows that PD-L1 mRNA is generally expressed by small percentages of GBM cells and macrophage/microglia (Figure 2B). There was no significant correlation between levels of CD3 positive cells and PD-L1 positivity (Pearson product moment correlation *P* = .274). PD-L1 expression was not associated with response (*P* = .78). Samples from second surgeries were available from four patients. Immunohistochemical data for these, together with the first surgery sample from the same patient, are shown in Figure 2C. Although there were small changes between first and second surgery samples, these were not consistent between patients and they cannot be definitively ascribed to avelumab treatment due to the small numbers of samples and the lack of a control arm in this study.

A recent report showed that in GBM there was no association between overall mutational burden and response to PD-1 inhibitors^33^; however, the same study showed that there was a link between *PTEN* status and response. Based on this, PTEN expression was assessed in the patients enrolled in this trial. Nine of 24 patients showed weak positive staining for PTEN in cancer cells, with the remainder showing no staining in cancer cells. None of the patients had staining with an intensity comparable to normal cells in the sample. Twenty-seven percent of nonresponders were weakly positive for PTEN, while 57% of responders were weakly positive for PTEN (*P* = .15). Weakly positive PTEN patients had higher CD3, CD8, and CD68 densities, although the differences were not significant (*P* = .136, .084, and .0587, respectively). PTEN expression did not change between first and second surgery samples.

## Discussion

Our patient population appeared comparable in terms of demographic and immune characteristics to previous GBM clinical trial population.^20^ As results from the study by Stupp et al.^1^ are used as benchmarks for comparison with our survival outcomes, it is important to point out some differences between that study and our study. In the study by Stupp et al., patients with a WHO status of 2 or better were enrolled prior to commencing combination TMZ/radiotherapy, whereas in our study patients were recruited with a Kanorfsky score of 70 or better within 3 weeks of having completed their combined TMZ/radiotherapy. The study by Stupp et al. did not consider *IDH1* or *MGMT* status; in addition, 15% of their patients did not have the histological diagnosis confirmed and of those that did, 8% were found to have a diagnosis other than GBM. Stupp et al. defined progression as an increase in tumor size by 25% or more on CT or MRI scans taken every 3 months, or a sustained increase in steroid dose, whereas our study used the iRANO criteria and all patients were followed by MRI except for one who had a pacemaker. These differences in patient population and study methodology need to be taken into consideration when making comparisons.

The safety and tolerability results in our GBM population did not reveal any new safety signal when compared to avelumab studies in other cancers or when compared to other immunotherapy trials in GBM.^34,35^ A median PFS of 9.7 months and OS of 15.3 months with at 12 and 24 months PFS of 40% and 17% and OS of 67% and 30% are not significantly different from Stupp et al. The objective response rate of 23% is better than what was seen with other immunotherapies in GBM.^20^ This may be explained by avelumab’s different mechanism of action and/or our different study design, with avelumab being used early in the disease course as an addition to standard therapy. The responders per the iRANO criteria showed a superior median OS of 30.4 months versus 12.8 months (*P* ≤ .0001). A higher incidence of *IDH1*+ and *MGMT*+ patients found in the responder group could explain some of the difference in survival outcomes. Alternative scheduling of avelumab, either immediately after initial surgeries or shortly before second surgeries, is potential future areas to explore. With the assays used here, none of the biological markers assessed in pretreatment tissue samples displayed any associated predictive value on clinical outcomes. On-treatment assessment of biomarkers might be more useful in identifying potential responders and understanding underlying immune mechanisms.^36^ Potential candidates could include nonspecific markers of neuronal loss such serum neurofilament light (NFl) or glial fibrillary acidic protein (GFAP). NFl is a neuron-enriched protein and GFAP is a cytoskeletal protein in astrocytes. Both have been shown to increase with acute neurological insult, neurodegenerative or neuroinflammatory diseases. Hepner et al. showed an association between higher serum NFl and GFAP levels and disease progression in patients with CNS tumors versus patients with stable disease.^37^ On-treatment measurements of serum cytokines such TGFβ, IL10, and interferon γ could be informative as to the ongoing immunotherapy’s impact on the systemic immune system.

The persistent use of steroids at months 1 and 2 provided early and clear identification of a subgroup with poorer prognosis with a median OS of 12.4 months versus 22.6 months. The Checkmate 143 study also found a negative prognostic value associated with baseline use of corticosteroids.^18^ Early steroid dependency identifies more symptomatic tumors. This may be the result of tumor location, size, and degree of accompanying vasogenic edema or a combination of the previous. The continued use of steroids however also attenuates the potential benefit of immunotherapy. It is of note that the baseline use, the total cumulative dose, or the total duration of steroid use were not associated with any prognostic value. Therefore, our study confirms what other studies have noted in that the continued use of steroids early on is associated with poorer survival either by identifying a characteristic of the tumor or by attenuating the immunotherapy or both. It highlights the need for alternative ways to control vasogenic edema when using immunotherapy and/or identifies a subgroup of patients with GBM that are either less responsive to standard therapy and/or have a worse prognosis.

With respect to the potential of avelumab to mediate ADCC or other antibody-mediated mechanisms of cell killing, this would be expected to reduce PD-L1 positive cell densities with treatment. In the four patients where first and second surgery samples were analyzed, there was no evidence for this. While this is not conclusive evidence for a lack of activity, given the small sample size, it does suggest that combination strategies to enhance this activity might be considered. The manageable safety profile demonstrated in this study also support exploration of combination strategies. GBM cells express CD47,^38^ an antiphagocytic signal that is frequently overexpressed in cancers.^39^ Combining avelumab with CD47 inhibition strategies,^40^ several of which are undergoing clinical testing, could be a promising future direction to enhance its ability to directly induce cell killing. Another approach could be to enhance the Th1 response of avelumab by combining it with an adjuvant such as the BCG vaccine.^41^ In common with antibodies targeting PD-1, avelumab on its own probably is not sufficient to overcome the multifaceted local and systemic immunosuppression associated with GBM. The manageable safety data demonstrated here also justify exploring other combinations with avelumab that are designed to enhance antigen presentation, promote immune cell recruitment, or inhibit additional immunosuppressive mechanisms.^42^

## Conclusions

Avelumab when used in addition to standard therapy appears safe and did not generate any GBM specific TEAE. Avelumab did not improve median OS when compared to the Stupp 2005 study. Other than the persistent use of steroids at months 1 and 2, no other baseline demographic characteristic or immunohistochemical marker was found to have any prognostic value on OS. MGMT+ was associated with a better PFS but not OS. Further studies of avelumab in combination with other agents are warranted in GBM.
